# Health-related quality of life after robotic surgery for endometrial cancer: a prospective longitudinal one-year follow-up study

**DOI:** 10.1007/s00404-023-06917-w

**Published:** 2023-01-25

**Authors:** Anna Lindfors, Stina Järvholm, Pernilla Dahm-Kähler

**Affiliations:** 1 Department of Obstetrics and Gynecology, Institute of Clinical Science, Sahlgrenska Academy, University of Gothenburg, 41345 Gothenburg, Sweden; 2 Department of Gynecology, Sahlgrenska University Hospital, Gothenburg, Sweden

**Keywords:** Quality of life, Mental health, Depression, Anxiety, Endometrial cancer, Robotic surgical procedures

## Abstract

**Purpose:**

This study aimed to explore how patients treated for endometrial cancer (EC) with robotic surgery are affected in symptoms of anxiety and depression and HRQoL in the long term.

**Methods:**

Women scheduled for primary robotic surgery for EC were included (*n* = 64), in this single-center study. Socioeconomic variables were obtained at baseline. The European Organization for Research and Treatment of Cancers Quality of Life Questionnaire Core 30 (QLQ-C30), its module for EC (EN24), the Generalized Anxiety Disorder Scale (GAD-7), and the Patient Health Questionnaire Depression Scale (PHQ-9) were followed prospectively from baseline to 2 weeks, 3 months and 1 year postoperatively.

**Results:**

The number of patients scoring above the clinical threshold for anxiety decreased from 17 (27.0%) at baseline to 4 (7.0%) at 2 weeks (*p* = 0.012). Depressive symptoms were reported in 20% of patients at baseline and did not change significantly during the one-year follow-up (*p* = 0.58). A significant decrease in Global health status was seen at 2 weeks (from 69.8 to 62.7; *p* = 0.048), with return to baseline levels after 3 months (68.5; *p* = 0.32) and stable at 1 year. Unemployment, low income, and adjuvant therapy correlated with lower Global health status at 3 months.

**Conclusion:**

The significant proportion of patients with anxiety symptoms preoperatively reduced prompt after surgery, while the proportion with depression remained constant, indicating that the primary treatment has no long-term negative effect on patients’ mental health. At 3 months, there is no obvious remaining negative impact on patients’ HRQoL, and these results are consistent after 1 year.

**Supplementary Information:**

The online version contains supplementary material available at 10.1007/s00404-023-06917-w.

## What does this study add to the clinical work

**Table:** 

Undergoing primary robotic surgery for endometrial cancer seem not to increase risk for depression and anxiety during a one-year follow-up. After primary surgery for endometrial cancer health-related quality of life is regained within three months, but certain characteristics may put women at increased risk for lower health-related quality of life.	

## Introduction

Health-related quality of life (HRQoL) and patient-reported outcomes are increasingly recognized as important parts of treatment evaluation and effectiveness, as it gives insight into patients’ experience of care and symptoms related to the cancer diagnosis and treatment [1, 2].

Endometrial cancer (EC) is the most common gynecological malignancy in the Western world. In Sweden, the median age at EC diagnosis is 70 years [3]. With a 5-year relative survival of 85% over all stages, many women are cured [4]. The gold standard for primary treatment of EC is surgery with hysterectomy and bilateral salpingo–oophorectomy, with additional lymph node dissection and/or omental resection, as staging procedure, followed by adjuvant therapy in accordance with risk classification protocols [5]. The current surgical techniques for EC, with increasing use of minimally invasive surgery and robotic-assisted laparoscopy in particular, offers rapid physical recovery with beneficial surgical outcomes [6].

However, little is known about patients’ long-term experiences after surgery [7]. Having a cancer diagnosis may result in psychological strain and affect HRQoL [8]. Both depression and anxiety are known complications of cancer treatment. There are studies focusing on HRQoL after robotic surgery for EC, but few have used a validated questionnaire or evaluated patients’ symptoms of depression and anxiety after treatment, and further assessments of socioeconomic variables are scarce [2, 9, 10]. Increased knowledge about HRQoL after treatment for EC is essential to enable the clinician to adequately inform patients and offer support and possibly adjust interventions.

The primary aim of this study was to prospectively explore how EC patients’ HRQoL and mental health changes after primary robotic surgery, during a one-year postoperative period. The secondary aim was to analyze how socioeconomic variables may be associated with HRQoL in this group of patients.

## Material and methods

### Study population

Patients scheduled for primary treatment with robotic surgery for EC from June 5th, 2019, to June 5th, 2020, at a tertiary hospital, Sahlgrenska University Hospital, were included. The hospital is serving the Western Sweden health care region (1.9 million inhabitants). Experienced gynecologic oncology surgeons perform robotic surgeries and the DaVinci^®^ system has been in use since 2010. Inclusion criteria were: 18 years or older, scheduled for primary surgery for EC, histologic confirmation of any subtype or grade of EC, and tumor clinically and radiologically limited to the uterus, FIGO stage I–II [11]. Exclusion criteria were non-proficient in Swedish, cognitively unable to fill in the questionnaires independently, and/or having received neo-adjuvant treatment (chemotherapy or radiation) prior to surgery. Patients who met the inclusion criteria and were scheduled to undergo robotic-assisted laparoscopic hysterectomy and bilateral salpingo–oophorectomy with or without lymph node dissection procedures were invited to participate in the study. Decision to perform a more extensive surgical procedure (sentinel lymph node dissection, systemic lymphadenectomy, or omental biopsy) was based on the risk classification score according to the national guidelines [3]. All patients received pre- and postoperative care at the Gynecologic Oncology Department at Sahlgrenska University Hospital.

### Data collection

Patients were included in the study at the gynecologic surgical oncology ward in conjunction with planning and preparation for the primary surgical treatment. Questionnaires regarding HRQoL were answered on paper before surgery (baseline), and again at 2 weeks, 3 months and 1 year postoperatively.

At baseline, the questionnaires were filled out at the ward; at follow-ups (2 weeks, 3 months and 1 year), the questionnaires were sent by mail and returned in prepaid envelopes. Clinical and demographic data were reported by the patients and collected at baseline before surgery. These included self-reported chronic diseases and former psychiatric problems. Socioeconomic variables reported by the patients were registered, including marital status, family and social situation, employment, and financial status. Monthly net income was grouped in three categories: low, middle, and high (Table 1). The definition of ‘low income’ was based on the Swedish Pension System, where a relatively low economic standard is categorized as 60% of the median national income, set to 12,000 SEK/month in their latest report from 2018 [12]. Patients who did not return the questionnaires had a reminder sent to them first by mail and then by phone. Data on tumor characteristics, surgical stage, and postoperative adjuvant chemo- or radiotherapy was collected from the medical records.

**Table 1:** Patient and tumor characteristics

	*n* = 64	
	*n*	%
Age (years), mean (SD)	67.8 (9.6)	
43–64	24	37.5
65–90	40	62.5
BMI (kg/m^2^), mean (SD)	28 (6.0)	
≥ 30	22	34.4
< 30	42	65.6
Education level		
Elementary school	8	12.5
Secondary school	17	26.6
College/university	32	50.0
Other	7	10.9
Occupation		
Unemployed	2	3.1
Employed	19	29.7
Self-employed	2	3.1
Retired	41	64.1
Country of birth		
Sweden	53	82.8
Other	11	17.2
Marital status		
Married/cohabiting	37	57.8
Single	14	21.9
Widowed	10	15.6
Other	3	4.7
Children		
Yes	56	87.5
No	8	12.5
Financial status^a^		
Low income (< 1440 USD/month)	17	26.6
Middle income (1440–3000 USD/month)	35	54.7
High income (> 3000 USD/month)	12	18.8
Chronic disease		
Yes	22	34.4
No	42	65.6
Previous worries about own mental health		
Yes	19	29.7
No	45	70.3
Primary treatment		
Surgery alone	43	67.2
Surgery + adjuvant therapy	21	32.8
Adjuvant therapy		
Chemo	21	32.8
Chemo + external radiation therapy	8	12.5
Lymph node procedure		
Sentinel lymph node	23	35.9
Systematic lymphadenectomy	28	43.8
FIGO stage		
IA	36	56.3
IB	12	18.8
II	3	4.7
III-IV	13	20.3
Histology		
Endometroid grade 1	25	39.1
Endometroid grade 2	27	42.2
Endometroid grade 3	5	7.8
Non-endometroid	7	10.9

*BMI* body mass index, *FIGO* Fédération Internationale de Gynécologie et d’Obstétrique (International Federation of Gynecology and Obstetrics), *n* number, *SD* standard deviation

^a^Predefined as net monthly income, where a low income is < 12 000 SEK (1440 USD, based on the December 2020 currency rate of 1 USD = 8.34 SEK), a middle income is 12 000–25 000 SEK (1440–3000 USD), and a high income, > 25 000 SEK (> 3000 USD)

### Questionnaires

Symptoms of depression were measured using the Swedish version of the 9-item Patient Health Questionnaire Depression Scale (PHQ-9) [13]. This questionnaire scores each of the nine depressive symptom criteria, with a total score ranging from 0 to 27. Symptoms of anxiety were measured using the Swedish version of Generalized Anxiety Disorder Scale (GAD-7), a seven-item measure with a total possible score of 21 [14]. The questionnaires have both been validated in Swedish and have good psychometric properties, with high internal consistency [15]. Cut points of 5, 10, and 15 represent mild, moderate, and severe symptom levels on both the PHQ-9 and the GAD-7. The cut point is commonly ≥ 10 for ‘clinically significant’ symptoms for each scale [15].

Health-related quality of life was measured with the European Organization for Research and Treatment of Cancer Quality of Life Questionnaire Core 30 (QLQ-C30) and its complementary EC-specific module, EN24 [16]. The questionnaires have been validated in Swedish and are considered to have good psychometric qualities [17, 18].

The QLQ-C30 includes a global health status (GHS) scale, five functional scales (physical, role, emotional, cognitive, and social), three symptom scales (fatigue, nausea/vomiting, and pain), and six single items (dyspnea, insomnia, appetite loss, constipation, diarrhea, and financial difficulties). The supplementary module EN24 has three functional scales (sexual interest, sexual activity, and sexual enjoyment), five symptom scales (lymphedema, urological, gastrointestinal, poor body image and vaginal), and five single items (pain in back and pelvis, tingling/numbness, muscular pain, hair loss, and taste change).

In total, the two questionnaires contain 54 items. Higher scores on functional scales and the GHS, including items related to sexuality, indicate better functioning and quality of life. By contrast, a higher score on the symptom scales/items indicates a higher level of symptoms.

### Data analysis and statistics

The data from the QLQ-C30 and QLQ-EN24 were transformed linearly to a 0–100 scale for each scale/symptom, in accordance with the scoring manual of the European Organization for Research and Treatment of Cancer Quality of Life Group [19]. Definition of minimally meaningful change has been performed according to recommendations in Cocks et al. for QLQ-C30, where it is recommended to adjust this threshold depending on what subscale is analyzed and direction of observed change [20]. Clinically meaningful changes are graded as small, medium, or large*.* For EN24, there is no detailed guideline available, and a clinically meaningful change has been set to ± 5 points on the scale. Changes from baseline in continuous variables of the QLQ-C30 and EN24, as well as the PHQ-9 and GAD-7, were compared over time using the Wilcoxon signed rank test, while Sign test was used for categorical variables. A univariable and stepwise multivariable linear regression analysis was performed at 3 months, with GHS as dependent variable. All tests were two-sided, with alpha set to 0.05. The linear regression is presented with beta values (95% confidence interval (CI)), *p* value, and *R*^2^. The software package SAS 9.4 (SAS Institute Inc., Cary, NC, USA) was used for the analyses.

## Results

**Fig. 1:**
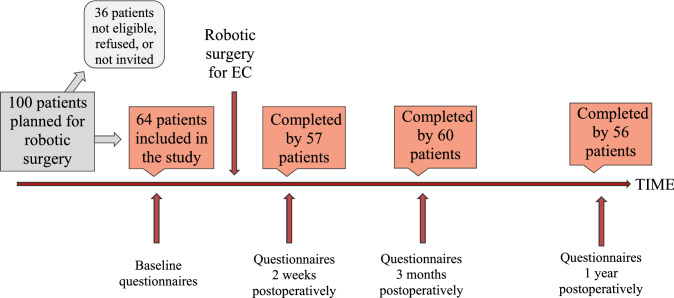
Flow chart of patients included in the study

During the study period, 100 patients with presumed stage I or II EC underwent primary robotic surgery. Out of these 64 agreed to participate and were included in the study, whereas 36 patients were not included due to exclusion criteria, not accepting, and giving consent, or organizational constraints resulting in individual patients not being offered participation, Fig. 1. Details on patient and tumor characteristics are presented in Table 1. The patients’ mean age was 67.8 years. Concerning socioeconomic variables, 37 (57.8%) patients were living in a relationship and the vast majority, 56 (87.5%), had children. Three patients (4.7%) were converted to laparotomy. Among the included patients, 43 (67.2%) were treated with primary surgery only, whereas 21 patients (32.8%) received adjuvant chemotherapy according to the national guidelines. Characteristics on patients possibly eligible but not included are presented in Supplementary Table 1 and show a higher mean age of 71.3 years, and a higher proportion of patients with Grade 3 or non-endometroid tumors, compared to the study population. Of the 64 patients included in the study, 57 (89.1%) answered the questionnaires at 2 weeks, 60 (93.8%) at 3 months and 56 (87.5%) patients completed the one-year follow-up (Fig. 1). Two patients died during the follow-up period.

### Depression and anxiety

**Fig. 2:**
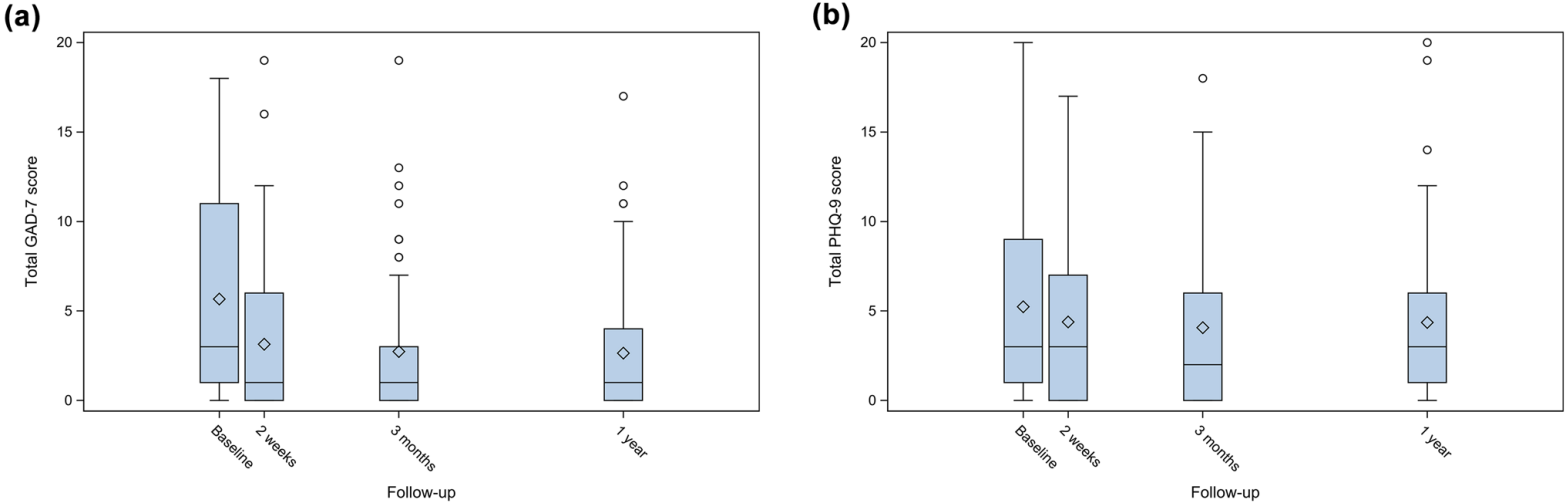
**a** Total Generalized Anxiety Disorder Scale (GAD-7) and **b** nine-item Patient Health Questionnaire Depression Scale (PHQ-9) scores at baseline, 2 weeks, 3 months and 1 year after robotic surgery

**Fig. 3:**
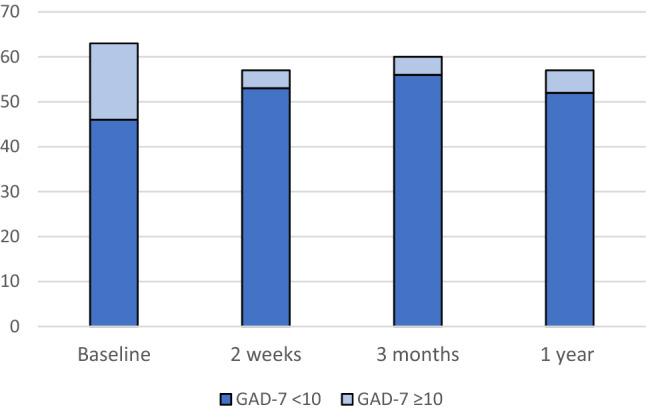
Number of patients scoring below and above the clinical threshold of  ≥ 10 on the General Anxiety Disorder Assessment (GAD-7) at different time points. The difference between baseline and the follow-up visits are all statistically significant

**Fig. 4:**
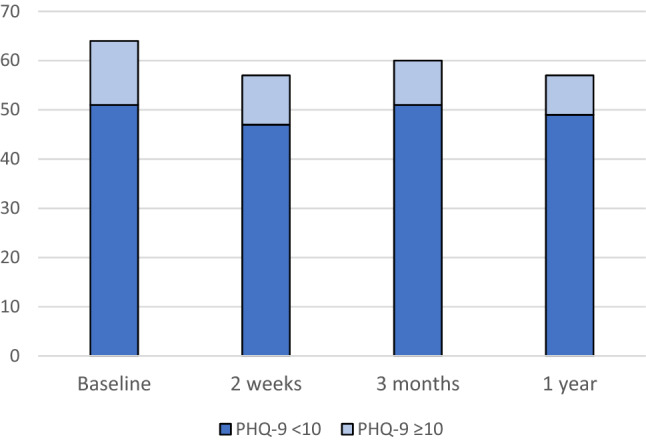
Number of patients scoring below and above the clinical threshold of  ≥ 10 on the Patient Health Questionnaire 9 (PHQ-9) at different time points. There is no statistically significant difference between the time points

Results for the GAD-7 and PHQ-9 are presented in Fig. 2a and b. The mean value for GAD-7 was 5.67 (standard deviation (SD) 5.67) at baseline, 3.14 (SD 4.30; *p* < 0.0001) at 2 weeks, 2.73 (SD 4.04; *p* < 0.0001) at 3 months and 2.64 (SD 3.75; *p* < 0.0001) at one-year follow-up. For PHQ-9, the corresponding values were 5.23 (SD 5.08), 4.39 (SD 4.72; *p* = 0.55), 4.07 (SD 4.63; *p* = 0.15) and 4.36 (SD 5.27; *p* = 0.58). The number of patients who reported above the clinical threshold for further assessment (≥ 10) on GAD-7 decreased significantly from baseline to 2 weeks and beyond, as illustrated in Fig. 3, from 17 (27.0%) at baseline to 4 (7.0%) at 2 weeks (*p* = 0.012) and remained at four (6.7%) at 3 months (*p* < 0.004) and five (8.9%) at 1 year (*p* = 0.035). The number of patients reporting above the clinical threshold (≥ 10) on the PHQ-9 was 13 (20.3%) at baseline, 10 (17.5%) at 2 weeks, 9 (15.0%) at 3 months and 8 (14.3%) at 1 year with no statistical significance difference (*p* = 0.55) (Fig. 4). Thirteen patients scored above the clinical threshold for depression at baseline. Twelve of these also did it for anxiety. Four of the five patients scoring above clinical threshold for anxiety at one-year follow-up, also did it for depression. For the sub-group receiving adjuvant therapy (*n* = 21) the number of patients reporting above the clinical threshold for further assessment (≥ 10) on GAD-7 were 5 (23.8%) at baseline, 1 (5.6%) at 2 weeks (*p* = 0.50), 1 (5.3%) at 3 months (*p* = 0.38) and 3 (16.7%) at one-year follow-up (*p* = 1.0). For PHQ-9, the corresponding values were 4 (19.0%), 3 (16.7%) (*p* = 1.0), 3 (15.8%) (*p* = 1.0) and 3 (16.7%) (*p* = 1.0).

None of the patients converted to open surgery (*n* = 3) ever scored above the clinical threshold for either anxiety or depression, except one patient at the one-year follow-up on GAD-7.

### The QLQ-C30 and EN24

Results from the QLQ-C30 and EN24 are presented in Table 2. Changes of importance met the criteria of both ‘clinically meaningful’ for each subscale, according to Cocks et al. [20], and statistically significant at the *p* < 0.05 level, and are summarized as follows: A small decrease was seen in GHS/quality of life after 2 weeks, with return to baseline levels after 3 months. For physical functioning, a small decrease, and for role functioning, a large decrease was seen at 2 weeks. At 3 months, both returned to baseline levels. Emotional and cognitive functioning were unchanged at 3 weeks but showed a medium and small improvement, respectively, at 3 months (Table 2). All changes seen between baseline and 3 months remained constant at 1 year. For the sub-group of patients receiving adjuvant therapy (*n* = 21) the mean GHS was 69.8 (SD 19.8) at baseline, 64.8 (SD 18.2); *p* = 0.22) at 2 weeks, 57.5 (SD 18.4; *p* = 0.016) at 3 months and 59.3 (SD 28.6; *p* = 0.28)) at one-year follow-up.

**Table 2:** Mean scores (standard deviation (SD)) for the European Organization for Research and Treatment of Cancers (EORTC) Quality of Life Questionnaire Core 30 (QLQ-C30) and endometrial cancer questionnaire EN24 from baseline to 2 weeks, 3 months and 1 year after robotic surgery

	Baseline *n* = 64	2 weeks *n* = 57	3 months *n* = 60	1 year *n* = 56	Change: baseline to 2 weeks	*p* value^a^	Change: baseline to 3 months	*p* value^a^	Change: baseline to 1 year	*p* value^a^
QLQ-C30										
Functional scales										
Global health status/QoL	69.8 (20.8) *n* = 64	62.7 (22.1) *n* = 57	68.5 (21.0) *n* = 60	69.2 (25.6) *n* = 56	− **8.77 (22.24)** *n* = 57	0.0048*	− 1.81 (22.40) *n* = 60	0.32	− 3.13 (28.05) *n* = 56	0.60
Physical functioning	82.0 (19.8) *n* = 64	71.3 (17.2) *n* = 56	80.1 (19.9) *n* = 60	80.8 (21.4) *n* = 56	− **12.4 (18.5)** *n* = 56	< 0.0001*	− 2.03 (10.85) *n* = 60	0.017*	− 2.59 (12.78) *n* = 56	0.21
Role functioning	78.6 (28.2) *n* = 64	52.4 (33.1) *n* = 56	79.1 (25.8) *n* = 59	82.1 (28.2) *n* = 56	− **30.1 (31.8)** *n* = 56	< 0.0001*	− 1.69 (26.75) *n* = 59	0.73	− 1.79 (24.76) *n* = 56	0.79
Emotional functioning	68.0 (26.1) *n* = 64	73.9 (20.7) *n* = 57	81.1 (20.3) *n* = 60	82.7 (20.5) *n* = 56	3.27 (21.09) *n* = 57	0.30	**12.3 (20.6)** *n* = 60	< 0.0001*	**9.92 (21.99) *n* = 56**	0.0001*
Cognitive functioning	79.9 (24.0) *n* = 64	83.0 (22.2) *n* = 57	85.8 (16.8) *n* = 60	88.1 (15.5) *n* = 56	1.46 (22.33) *n* = 57	0.60	**4.72** (17.38) *n* = 60	0.042*	4.17 (19.91) *n* = 56	0.18
Social functioning	84.6 (24.7) *n* = 64	79.8 (26.1) *n* = 57	85.0 (20.5) *n* = 60	84.8 (24.3) *n* = 56	− 6.43 (30.50) *n* = 57	0.10	-1.67 (19.58) *n* = 60	0.62	− 4.46 (27.43) *n* = 56	0.26
Symptom scales										
Fatigue	30.3 (23.8) *n* = 64	41.0 (24.0) *n* = 57	32.5 (26.6) *n* = 60	29.0 (27.7) *n* = 56	**12.7 (23.4)** *n* = 57	< 0.0001*	3.80 (22.79) *n* = 60	0.28	2.89 (17.66) *n* = 56	0.29
Nausea/vomiting	4.17 (8.91) *n* = 64	7.31 (12.21) *n* = 57	5.00 (13.48) *n* = 60	5.06 (13.07) *n* = 56	3.22 (13.52) *n* = 57	0.11	1.67 (13.62) *n* = 60	0.48	1.49 (13.95) *n* = 56	0.46
Pain	17.7 (25.5) *n* = 64	33.0 (27.9) *n* = 57	20.0 (28.1) *n* = 60	22.3 (30.4) *n* = 56	**17.3 (22.7)** *n* = 57	< 0.0001*	4.17 (23.29) *n* = 60	0.10	**8.93 (26.96) *n* = 56**	0.018*
Dyspnea	15.9 (21.5) *n* = 63	21.4 (23.3) *n* = 56	22.2 (22.7) *n* = 60	22.6 (25.5) *n* = 56	**7.27 (24.59)** *n* = 55	0.029*	**6.78 (22.98)** *n* = 59	0.041*	**7.88 (23.97) *n* = 55**	0.026*
Insomnia	29.2 (33.3) *n* = 64	29.8 (31.3) *n* = 57	28.3 (25.9) *n* = 60	25.0 (29.3) *n* = 56	1.75 (34.17) *n* = 57	0.66	1.11 (28.10) *n* = 60	0.79	− 1.19 (24.59) *n* = 56	0.72
Appetite loss	10.4 (23.7) *n* = 64	16.1 (23.8) *n* = 56	9.44 (22.21) *n* = 60	8.93 (22.47) *n* = 56	7.14 (31.60) *n* = 56	0.077	0.556 (23.363) *n* = 60	0.95	2.98 (25.64) *n* = 56	0.41
Constipation	12.5 (25.5) *n* = 64	22.8 (31.0) *n* = 57	20.0 (30.2) *n* = 60	17.9 (29.8) *n* = 56	**10.5 (37.4)** *n* = 57	0.037*	**7.22 (23.84)** *n* = 60	0.026*	5.36 (20.87) *n* = 56	0.093
Diarrhea	6.77 (17.99) *n* = 64	12.3 (25.7) *n* = 57	10.00 (20.63) *n* = 60	10.7 (25.5) *n* = 56	7.02 (24.99) *n* = 57	0.055	5.00 (19.24) *n* = 60	0.068	5.95 (23.87) *n* = 56	0.10
Financial problems	7.81 (22.80) *n* = 64	7.60 (17.84) *n* = 57	5.56 (15.24) *n* = 60	5.36 (15.28) *n* = 56	0.000 (23.570) *n* = 57	0.98	0.000 (20.355) *n* = 60	1.00	− 0.00 (22.92) *n* = 56	1.00
EN24										
Functional scales										
Sexual interest	11.3 (21.7) *n* = 62		14.3 (25.3) *n* = 56	19.0 (25.3) *n* = 56			5.45 (22.00) *n* = 55	0.11	**10.5 (24.9) *n* = 54**	0.0039*
Sexual activity	9.14 (20.17) *n* = 62		12.5 (20.7) *n* = 56	16.7 (24.6) *n* = 56			4.85 (22.61) *n* = 55	0.17	**9.26 (23.72) *n* = 54**	0.0072*
Sexual enjoyment	69.7 (27.7) *n* = 11		64.3 (27.6) *n* = 14	50.0 (29.6) *n* = 20			0.000 (27.217) *n* = 7	1.00	− 12.5 (24.8) *n* = 8	0.38
Symptom scales										
Lymphedema	10.2 (18.0) *n* = 64	8.48 (18.13) *n* = 57	16.7 (21.0) *n* = 60	17.0 (25.1) *n* = 56	− 0.877 (14.578) *n* = 57	0.66	**6.94 (16.61)** *n* = 60	0.0018*	**8.33 (22.47) *n* = 56**	0.0025*
Urological symptoms	22.0 (21.4) *n* = 64	13.4 (13.6) *n* = 57	20.0 (22.0) *n* = 60	20.8 (23.9) *n* = 56	− **6.97 (20.79)** *n* = 57	0.024*	-1.53 (16.81) *n* = 60	0.50	− 0.595 (20.106) *n* = 56	0.72
Gastrointestinal symptoms	18.4 (18.4) *n* = 64	29.6 (19.6) *n* = 57	19.1 (14.6) *n* = 60	19.8 (21.2) *n* = 56	**13.0 (16.9)** *n* = 57	< 0.0001*	1.58 (14.73) *n* = 60	0.19	3.27 (18.22) *n* = 56	0.28
Poor body Image	15.9 (27.3) *n* = 64	11.9 (19.3) *n* = 56	16.7 (27.3) *n* = 60	13.4 (25.1) *n* = 56	− 0.298 (23.245) *n* = 56	0.72	2.50 (33.02) *n* = 60	0.54	0.893 (30.221) *n* = 56	0.80
Sexual/vaginal problems	16.2 (29.5) *n* = 11		25.4 (28.4) *n* = 14	20.6 (23.6) *n* = 21			3.17 (26.23)	0.75	1.39 (35.85) *n* = 55	0.66
Pain in back and pelvis	30.6 (32.1) *n* = 62	23.2 (24.6) *n* = 56	27.0 (31.5) *n* = 58	32.1 (34.2) *n* = 56	− 6.79 (29.23) *n* = 54	0.10	− 4.09 (28.22) *n* = 57	0.29	4.24 (30.80) *n* = 55	0.35
Tingling/numbness	13.0 (24.2) *n* = 64	4.76 (13.38) *n* = 56	21.5 (31.4) *n* = 59	28.6 (33.9) *n* = 56	− 4.76 (23.29) *n* = 56	0.19	**10.7 (30.0)** *n* = 59	0.0059*	**18.5 (33.6) *n* = 56**	< 0.0001*
Muscular pain	31.3 (33.5) *n* = 64	19.3 (26.7) *n* = 57	29.4 (32.5) *n* = 60	32.7 (37.3) *n* = 56	**− 9.94 (29.52)** *n* = 57	0.0096*	0.000 (29.433) *n* = 60	0.98	6.55 (35.63) *n* = 56	0.18
Hair loss	12.2 (23.4) *n* = 63	2.34 (13.89) *n* = 57	35.0 (41.8) *n* = 60	18.5 (29.8) *n* = 56	**− 7.14 (16.47)** *n* = 56	0.0032*	**23.7 (42.9)** *n* = 59	< 0.0001*	**8.93 (31.46) *n* = 56**	0.028*
Taste change	8.33 (22.22) *n* = 64	13.5 (23.5) *n* = 57	16.7 (29.8) *n* = 60	9.52 (24.38) *n* = 56	**6.43 (25.54)** *n* = 57	0.042*	**8.33 (27.87)** *n* = 60	0.032*	2.98 (21.35) *n* = 56	0.34

Raw scores converted to 0–100 scale. Values in mean (SD). Values in bold represent a clinically meaningful and statistically significant change

*QoL* quality of life

*Statistically significant at the 5% level

^a^For comparison over time, the Wilcoxon signed rank test was used

### Regression analysis

**Fig. 5:**
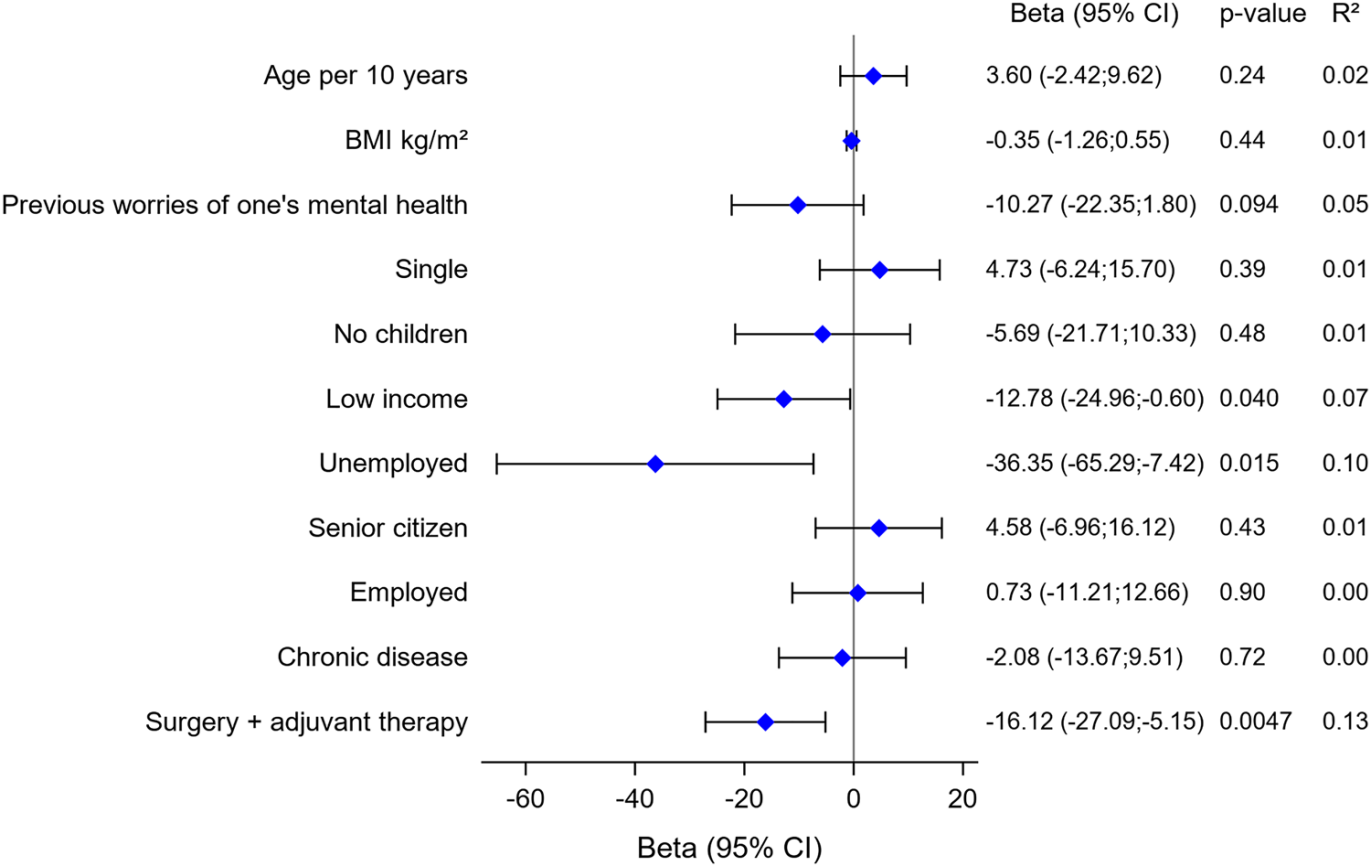
Univariate linear regression of correlation between patient characteristics and global health status (GHS) at 3 months. Beta values and 95% confidence intervals (CIs) are shown

Figure 5 presents a forest plot of correlation between variables of patient characteristics and GHS at 3 months, estimated by univariable analysis. Unemployment, low income, and receiving adjuvant therapy were all statistically significantly negatively correlated with GHS.

## Discussion

Questions regarding HRQoL and psychological wellbeing are an essential part of care for cancer patients. Increased interest from caregivers and patients has been seen, but still, patients’ experiences of primary treatment for EC are not adequately explored. In this study, we present patients’ symptoms of anxiety and depression and HRQoL during 1 year after robotic surgery for EC, together with associations with patient socioeconomic characteristics. Our main findings indicate that the primary treatment has no long-term negative effect on symptoms of depression and anxiety and HRQoL returns to baseline levels 3 months after surgery. However, certain characteristics may put patients at increased risk of lower HRQoL.

The participants mean scores were comparable to the norm population for GAD-7 and PHQ-9 throughout the follow-up [21]. Despite this, a significant proportion of patients scored above the threshold for clinically significant symptoms of anxiety, 17 (27%), and depression, 13 (20.3%), at baseline. This can be compared with the general population where clinically significant anxiety (≥ 8) was found to have a prevalence of 17.9% (95% CI 15.1–20.7) and the corresponding prevalence of clinically significant depression was 12.9% (95% CI 10.4–15.4) among Swedish women [21]. When baseline measures were registered, patients had received information about their diagnosis but had not yet undergone surgery. This period of stress and uncertainty is likely to affect these baseline data. Fortunately, significantly fewer patients scored ≥ 10 on the GAD-7 at all time points during follow-up, strengthening the theory that the increased anxiety was related to upcoming surgery and receiving a cancer diagnosis. Furthermore, this may be emphasized by the increase seen in emotional and cognitive functioning at 3 months in the QLQ-C30. Most patients reaching the clinical threshold for anxiety simultaneously reached it for depression, indicating prevalent comorbidity in this group of women which is a known phenomenon in the general population.

Notably, the numbers of patients scoring ≥ 10 on the PHQ-9 is fairly stable during the time of follow-up. An Italian study assessing symptoms of anxiety and depression longitudinally in patients undergoing surgery for EC reported similar findings, with symptoms of anxiety decreasing over time but those of depression staying stable [10].

The pattern of an initial decrease in GHS after surgery, followed by a return to baseline values, may be expected due to the obvious physical impact of the surgery, but also due to emotional distress associated with the diagnosis and time spent in hospital [8, 22]. A Danish study by Herling et al. uses the same questionnaires as used in our study and reports a similar development, with GHS at their second follow-up at 5 weeks already increased beyond baseline values [23]. Interestingly, patients’ GHS before diagnosis is not known, but in a large, systematic study on norm data in the general population with a cohort of 15 386 men and women across Europe and North America, mean GHS was found to be 69.2 (SD 22.1) in the Swedish population and 64.3 (SD 21.8) in all European women [24]. This is reassuring in regard to our results, with GHS of 69.8 at baseline and 69.2 after 1 year, indicating that neither the diagnosis nor the surgery compromised the GHS.

A large decrease in role functioning was seen in this study at 2 weeks, but with a return to baseline levels at 3 months. This may be explained by the surgical trauma affecting physical function and possible sick leave. Though not using the exact same time points for follow-up, a similar pattern was described by Herling et al. [23].

The regression analysis identified the variables, receiving adjuvant therapy, being unemployed and having a low income as risk factors for reduced GHS. The analysis is, however, based on few patients, since only two women reported unemployment, which should be taken into consideration. Nevertheless, socioeconomic disparities are known risk factors for impaired HRQoL, which should possibly also receive attention in this patient group [10, 25, 26]. More specifically a Norwegian study showed that survivors of gynecological cancer with low income more often suffer from pain and impaired HRQoL [27].

Taken together, our results indicate that symptoms of depression and anxiety, as seen among other cancer patients, is not a prevalent issue in this group of EC patients. On the contrary, they seem to withstand the treatment and post-treatment phase well and report a psychological wellbeing and HRQoL on a level comparable to the norm population in a long-term follow-up.

Strengths of our study are the prospective longitudinal long-term follow-up, with high retention and precise time points for assessment. The use of validated, internationally known questionnaires increases the quality of patient-reported outcomes and offers an opportunity to make comparisons between different patient populations. This study is, to our knowledge, unique in assessing symptoms of depression and anxiety in EC patients using the GAD-7 and PHQ-9. Limitations include the study’s single-center design, affecting external validity. Another drawback is the heterogeneity of the cohort, with 32.8% receiving adjuvant therapy, which may negatively affect the internal validity. Not only the negative side effects but possibly also the fact that these patients are identified as risk group for recurrent disease, may affect their psychological wellbeing [8, 28]. The sub-analysis of the patients receiving adjuvant therapy indicates a trend towards lower GHS at 3 months and 1 year, which was also evident in the regression analysis. Moreover, the limited information about patients not included hampers an analysis of possible systematic differences between these and the studied individuals, and hence constitutes a possible selection bias. The higher mean age among those not included, may be an expression of older patients being included in clinical trials less often, a phenomenon seen widely. Further, the lower rates of lymphadenectomy and adjuvant therapy among the not included patients, is possibly partly a result of their higher age. This could subsequently have a positive impact on HRQoL and mental health in this group compared to the study population, and hence bias our results in the negative direction. Longitudinal studies of patient-reported outcomes have inherent problems of response shift over time, which is difficult to control. When asking study patients, the identical question later, the former problem may not appear as troublesome; however, it is the patients’ experience we aim to capture, and this may illustrate their coping capacity. Adding a qualitative approach, with gaining in-depth knowledge about women’s own experiences, would have given increased insight.

## Conclusions

This study adds valuable knowledge about long-term HRQoL and mental health in EC patients undergoing robotic surgical primary treatment. The significant proportion of patients with anxiety symptoms preoperatively reduced prompt after surgery below norm levels, while the proportion with depression remained stable, indicating that the primary treatment has no long-term negative effect on patients’ mental health in this cohort. At 3 months follow-up, there is no obvious remaining negative impact on patients’ HRQoL, and these results are consistent after 1 year. However, being unemployed, receiving adjuvant therapy, and having a low income may put patients at risk of impaired HRQoL.

## Supplementary Information

Below is the link to the electronic supplementary material.

Supplementary file1 (XLSX 10 KB)

## Data Availability

The data that support the findings of this study can be available on reasonable request from the corresponding author, [AL].
